# On the Use of Log Wood in Cancer

**Published:** 1862-12

**Authors:** 


*On the use of Log-wood in Cancer.*—M. Desmartis lately brought under the notice of the Academy the results obtained by him with the extract of log-wood applied to cancerous ulcers. He found that by the use of an ointment composed of equal parts of fat and extract of log-wood, not only the odor disappeared, which was in some instances of the most offensive description, but that the suppuration was also considerably diminished. As soon as this ointment was again discontinued, the odor reappeared, and the secretion assumed an unfavorable character. In cases of hospital gangrene, the same ointment acts like a charm, and it likewise proves valuable in erysipelas consequent upon amputations and wounds, even if the form of the disease is most severe. Log-wood may be mixed with styptics, such as ergot of rye, perchloride of iron, persulphate of iron, and employed as powder or as lotion. The extract is only soluble in hot water, and its price is merely nominal.—*Medical Times.*

